# Wrist movements induce torque and lever force in the scaphoid: an ex vivo study

**DOI:** 10.1186/s13018-020-01897-y

**Published:** 2020-08-31

**Authors:** Jochen Erhart, Ewald Unger, Philip Schefzig, Peter Varga, Michael Hagmann, Robin Ristl, Stefan Hajdu, Anna Gormasz, Patrick Sadoghi, Winfried Mayr

**Affiliations:** 1 Department of Trauma Surgery, Medical University of Vienna, Währinger Gürtel 18-20, A-1090 Vienna, Austria; 2 Center for Medical Physics and Biomedical Engineering, Medical University of Vienna, Währinger Gürtel 18-20, A-1090 Vienna, Austria; 3 AO Foundation, Clavadelerstrasse 8, 7270 Davos, Switzerland; 4 Core Unit of Medical Statistics Medical Statistics and Informatics, Spitalgasse 23, BT88 University of Vienna, A-1090 Vienna, Austria; 5 Department of Orthopedic and Trauma Surgery, Medical University Graz, Graz, Austria

**Keywords:** Scaphoid fracture, Biomechanics, Wrist movement, Torque and lever force

## Abstract

**Purpose:**

We hypothesised that intercarpal K-wire fixation of adjacent carpal bones would reduce torque and lever force within a fractured scaphoid bone.

**Methods:**

In eight cadaver wrists, a scaphoid osteotomy was stabilised using a locking nail, which also functioned as a sensor to measure isometric torque and lever forces between the fragments. The wrist was moved through 80% of full range of motion (ROM) to generate torque and force within the scaphoid. Testing was performed with and without loading of the wrist and K-wire stabilisation of the adjacent carpal bones.

**Results:**

Average torque and lever force values were 49.6 ± 25.1 Nmm and 3.5 ± 0.9 N during extension and 41 ± 26.7 Nmm and 8.1 ± 2.8 N during flexion. Torque and lever force did not depend on scaphoid size, individual wrist ROM, or deviations of the sensor versus the anatomic axis. K-wire fixation did not produce significant changes in average torque and lever force values except with wrist radial abduction (*P* = 0.0485). Other than wrist extension, torque direction was not predictable.

**Conclusion:**

In unstable scaphoid fractures, we suggest securing rotational stability with selected implants for functional postoperative care. Wrist ROM within 20% extension and radial abduction to 50% flexion limit torque and lever force exacerbation between scaphoid fragments.

## Introduction

The scaphoid is the most frequently fractured bone in the wrist and presents clinical challenges that include inadequate diagnosis as well as healing. Among these, vascular supply has been viewed as the main reason for problems in treating scaphoid fractures and non-unions [1]. Recent anatomical studies have demonstrated that difficulties in scaphoid bone healing cannot be attributed solely to vascularisation [2]. The fractured scaphoid causes stability problems in its dual function as a force transmitter and simultaneous coordinator of movements in adjacent wrist bones [3]. The most important forces involved in micro-motion at the fracture site are bending and shear forces [4]. The efficiency of single-screw osteosyntheses, in particular in fractures and non-unions of the scaphoid bone, has not been thoroughly analysed. Providing the best possible fracture stabilisation and postoperative treatment management requires considering the direction and magnitude of isometric torque and lever force within the scaphoid bone [5, 6]. Postoperative functional therapy requires knowing the dimension and direction of the torque through the scaphoid during different wrist movements. Therefore, the primary aim of this study was to measure the magnitude and direction of induced isometric torque and lever force in a reconnected osteotomy gap of the scaphoid. These measurements were made in cadaver forearms with varying magnitudes and directions of wrist motion and a variety of preload conditions. The secondary study aim was to examine the influence of systematic parameters (scaphoid shape and size, deviation between the sensor axis after implantation relative the anatomic longitudinal axis of the scaphoid bone, maximum potential wrist range of motion [ROM]) on the amount of torque. Our hypothesis was that intercarpal K-wire fixation of the adjacent carpal bones would reduce torque and lever force within the fractured scaphoid bone.

## Materials and methods

The wrists of eight anonymous cadaver forearms (body donors from the local anatomy institute) were passively moved in four directions (flexion, extension, radial abduction, ulnar duction) following scaphoid osteotomy and reconnection with an implanted isometric sensor. The resulting change in torque relative to wrist positioning was measured. The methodology, in particular surgical technique, sensor structure, force distribution, and forearm fixation, was developed in a pilot study and recently published [7].

**Fig. 1:**
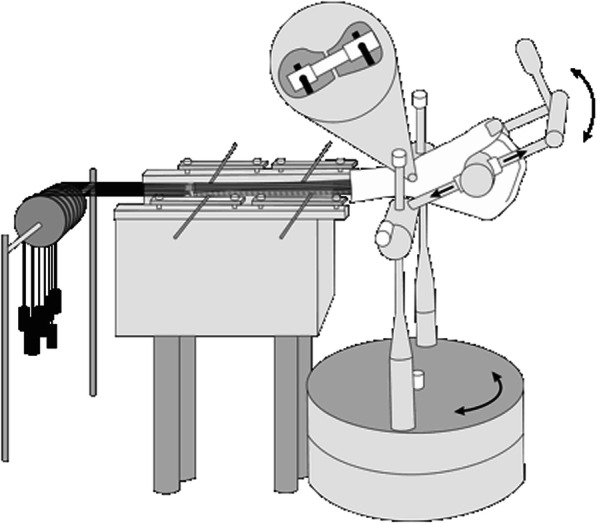
The testing setup with fastening of the forearm, retaining the ability to produce wrist movements. Loading of the wrist was achieved by weights attached to a pulley system, generating tendon loading. The figure shows an enlarged version of the sensor located in the osteotomised scaphoid that measured rotation and torque

**Fig. 2:**
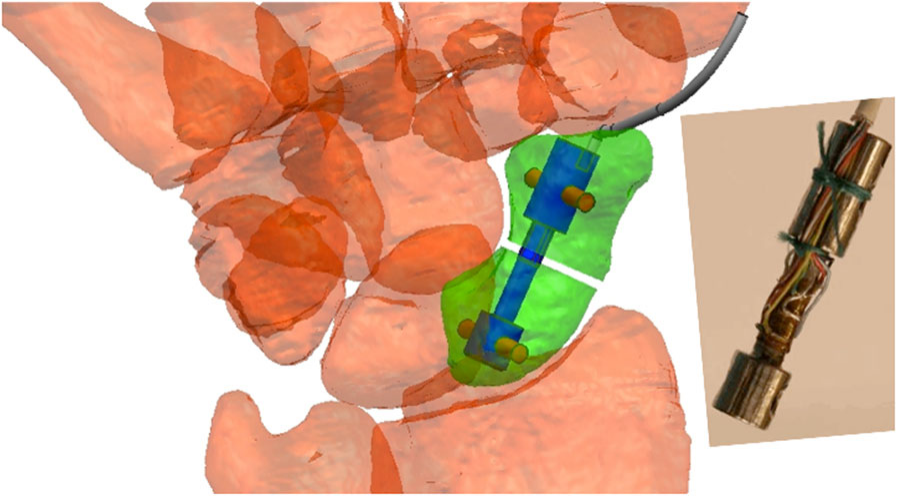
Photo of the sensor and schematic drawing of the sensor that was implanted and interlocked within the osteotomised scaphoid. The position of strain gauges on the sensor and draft of Wheatstone bridge circuits have been published previously [7]

**Fig. 3:**
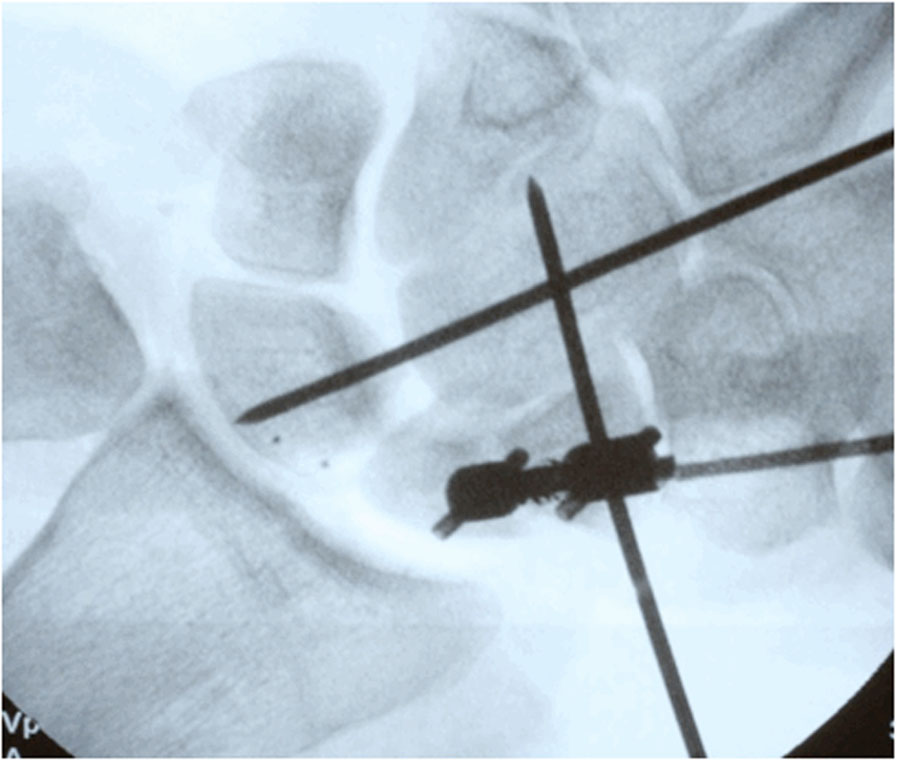
X-ray of the experimental wrist setup. The fixation locking nail was used as a sensor between the two scaphoid fragments. In addition, the midcarpal joint was partially stabilised with K-wires

The setup in this investigation consisted of a forearm and a mobile hand fixation system with an integrated goniometer to record angles of wrist movement in two orthogonal planes. In addition, the system allowed for preloading the wrist (Table 1) using a set of weights fixed to the main hand tendons (Fig. 1). Part of the experimental setup was an implantable sensor that performed like a scaphoid screw and could sense torque strain by a Wheatstone full-bridge and force strain by a Wheatstone half-bridge between the two bone fragments (Figs. 2 and 3) [7]. To carry out the measurements, we fixed the forearm on a block with four half-pins to allow a grasping movement and unhindered movement of the wrist. To simulate a loaded wrist, 16 lower arm tendons were assembled into seven dominant force cords and guided with pulleys. These tendons were pulled with enough force to mimic a co-contraction of the wrist and to form a fist. Identical loading of tendons was performed in each specimen. To prevent uncontrolled movements of the wrist, we bandaged the metacarpus to a holder that was mobile in the axial plane. Possible rotation in the coronal and sagittal planes could be blocked individually. The experimental setup allowed placement of the specimen in a neutral position for the forearm and wrist. This neutral alignment was defined as the starting point for further measurements and positions.

**Table 1:** Substitution of muscle forces in the biomechanical setup by weight loading of tendons and tendon groups

Number of tendons	Tendon group	Substitution weight
1	Flexor carpi radialis	10 N
1	Flexor pollicis longus	5 N
8	Flexor digitorum superficialis and profundus	5 N
1	Flexor carpi ulnaris	10 N
2	Extensor pollicis brevis and abductor pollicis longus	10 N
1	Extensor carpi ulnaris	10 N
2	Extensor carpi radialis (longus and brevis)	20 N

Seven tendon groups causing a grasping position of the hand and wrist stabilisation

**Fig. 4:**
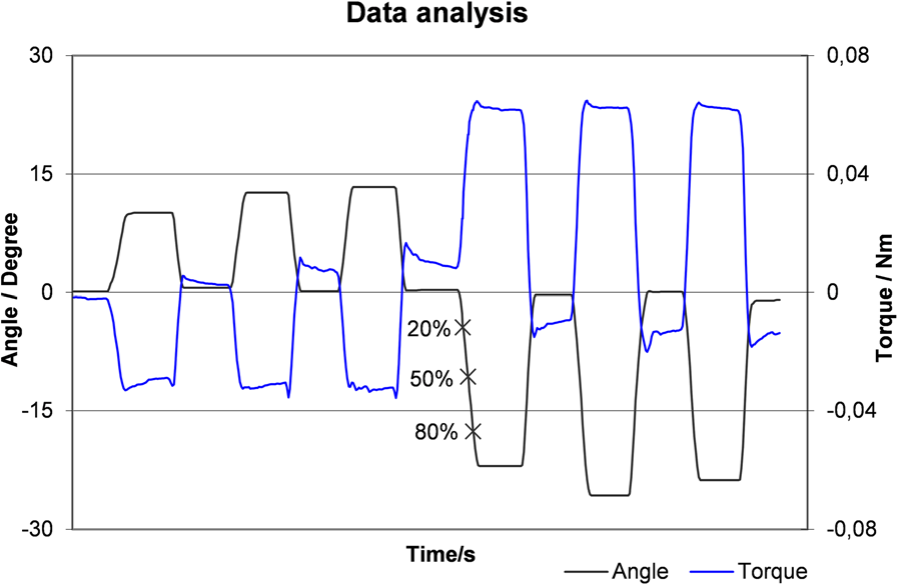
Representation of torque at 20%, 50%, and 80% of ROM in radial and ulnar directions of the wrist. Using MATLAB (MathWorks, Adalperostraße, Ismaning, Germany), digital goniometer-based ROM, torque, and two simultaneously recorded forces were collected over time

Guided movement of the wrist was performed with and without joint loading in all four directions. Angle of the wrist movement sequences and torque within the scaphoid were continuously recorded. The setup allowed guidance of uniaxial wrist movements and prevented deviations in other directions. Each individual movement cycle was repeated three times and controlled manually by an operator until resistance indicated the end of the wrist’s ROM. To exclude interobserver variability, a single operator carried out all measurements for maximal ROM. As a way to avoid excessive tension towards the end of the ROM, repetitive movement cycles were evaluated only up to 80% of the entire ROM. Figure 4 shows the graphic evaluation using MATLAB (MathWorks, Ismaning, Germany).

To measure the biomechanical interaction between the two fracture segments of the osteotomised scaphoid, we developed a sensor that also served as a rigidly binding osteosynthesis implant (Figs. 2 and 3). This sensor has been described in detail previously [7]. In this study, the system was used to record the torque and the goniometer signal. It also transferred all recorded data digitally to a PC system in digital format at a sampling rate of 100 Hz. The components of force in the *x* and *y* directions were not recorded for this investigation. A standardised osteotomy of the scaphoid was performed on the palmar side in a lateral direction within the middle third of the scaphoid. The capsulotomy required for the osteotomy was closed with Ethibond® suture (Ethicon, West Somerville, NJ).

To further stabilise the scaphoid fragments, we introduced two K-wires from the distal scaphoid fragment into the capitate and from the capitate into the lunate (Fig. 3). Afterward, we measured guided movements under the following four conditions: (1) no loading without K-wires, (2) loading without K-wires, (3) no loading with K-wires, and (4) loading with K-wires.

Following sensor explantation, we documented the position of the drilling canals within the scaphoid fragments used high-resolution peripheral quantitative computed tomography (SCANCO Medical, Bruettisellen, Switzerland). With this tool, we analysed the position of the sensor and the osteotomy within the scaphoid bone. After these measurements, the position and quality of the implanted sensor and condition of the ligaments were examined during dissection. The cadaver wrists were examined for arthritis and an intact ligamentous structure after the experiment was conducted. Loosening of the sensor was ruled out by dissection following testing. Hand and scaphoid sizes varied considerably, so we used water displacement to measure the scaphoid volume. Because of the small dimension, incompletely transected scaphoids and transection of a sensor cable forced us to abandon six preparations, and we had to process 14 cadaver hands to obtain a sample size of 8.

### Statistical evaluation

Absolute torque and force were measured at 20%, 50%, and 80% of total ROM in each wrist movement direction (extension, flexion, radial abduction, and ulnar abduction) and for each combination of the two experimental factors K-wire arthrodesis (with K-wire vs without K-wire) and wrist loading (loaded vs unloaded). All measurements were repeated four times. Prior to the statistical analysis, these four repeated measurements were averaged to obtain a single torque or force value under each experimental condition. The means and standard deviations of torque and force were calculated for each experimental condition.

To further analyse the effect of K-wire arthrodesis and wrist loading on torque and force, we fit separate linear mixed models for force and torque values obtained under a given combination of total ROM and wrist movement direction. The models included the factors K-wire arthrodesis, wrist loading, and their interaction as explanatory variables. Scaphoid identity was included as a random factor to account for the dependency between observations of the same scaphoid under different experimental conditions. From these models, we calculated the mean differences between the two levels of each factor (averaging across the levels of the other factor). The null hypothesis of a true mean difference of zero was tested using an *F*-test. To account for performing 48 hypothesis tests in total, Bonferroni–Holm-adjusted *P* values are reported in addition to unadjusted *P* values. We also calculated *P* values for the null hypothesis of no interaction between the factors K-wire and wrist loading. We viewed these tests as exploratory and did not include them in the multiple testing adjustment.

All experiments were carried out in accordance with relevant guidelines and regulations. This study was approved by the local institutional ethics board (Ethics Approval No.: 785/2008; date: January 13, 2009).

## Results

**Fig. 5:**
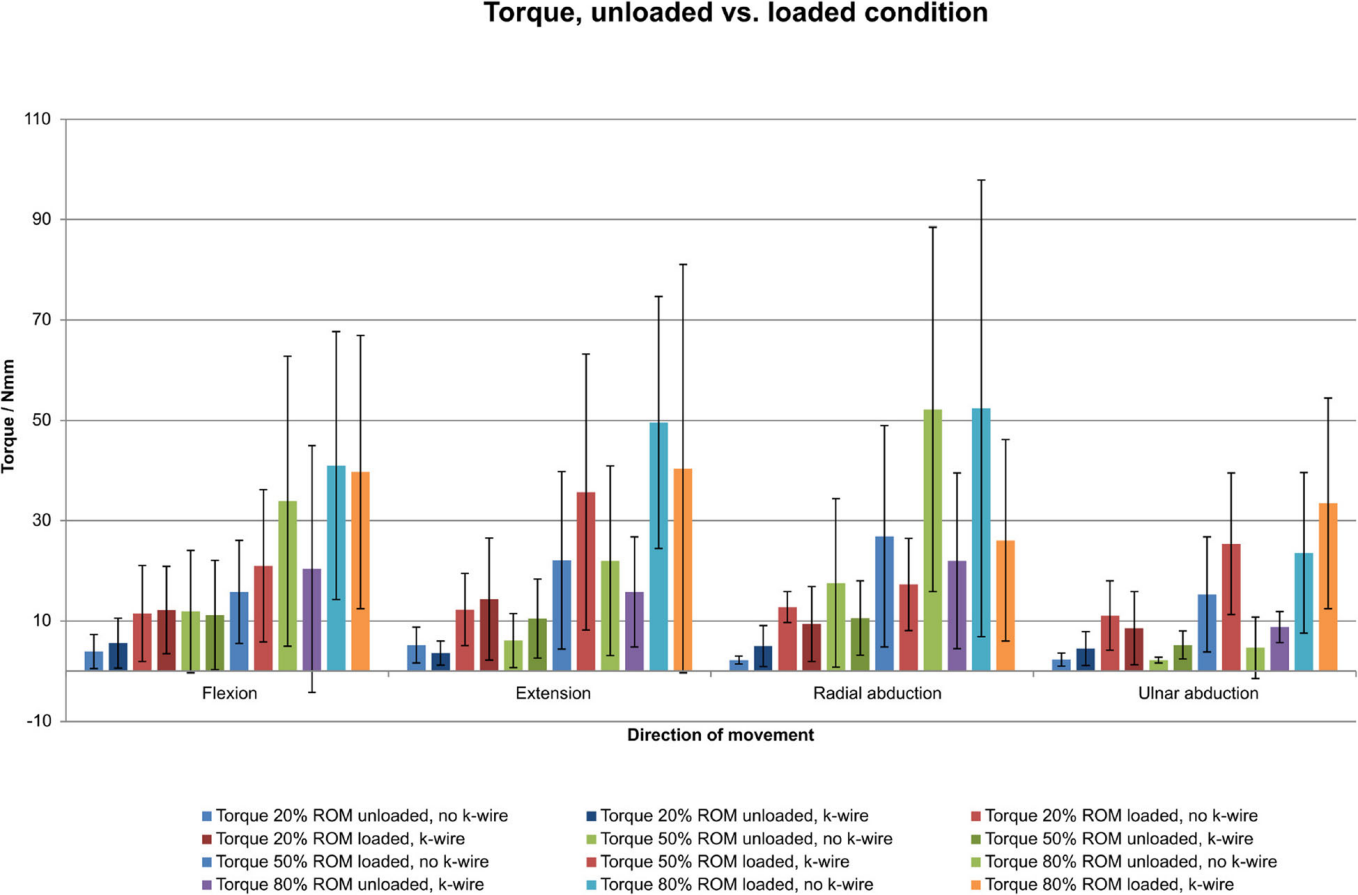
Torque within the scaphoid at 20%, 50%, and 80% of wrist ROM (flexion, extension, radial abduction, ulnar abduction) in the unloaded and loaded states with and without K-wire stabilisation

Wrist movement in all four main directions caused measurable torque and force within the osteotomised scaphoid bone (Table 2). The K-wire arthrodesis showed no significant reduction in torque and force (adjusted *P* values from 0.0569 to 1.000) in any model but did so in torque through 80% radial duction (adjusted *P* value, 0.0485) (see Table 2 for details). Tendon loading was the only factor that significantly affected torque and force in every wrist direction but not every ROM (Table 2, Fig. 5, and Supplementary Figure 1).

**Table 2:** Sample mean (standard deviation) under different experimental conditions

Dir.	y_name	ROM	unloaded,no k-wire	unloaded,k-wire	loaded,no k-wire	loaded,k-wire	Wire difference	Wire *P*	Wire *P*-adjusted	Loading difference	Loading *P*	Loading *P*-adjusted	Interaction *P*
F	Torque	20	3.9 (3.4)	5.6 (5)	11.5 (9.6)	12.2 (8.7)	1.2 [− 2.3, 4.7]	0.4897	1	7.1 [3.5, 10.6]	0.0004	0.0172	0.769
F	Torque	50	11.9 (12.2)	11.2 (10.9)	15.8 (10.3)	21 (15.2)	2.2 [− 5.7, 10.1]	0.5622	1	6.8 [− 1.1, 14.7]	0.0885	1	0.4487
F	Torque	80	33.9 (28.9)	20.4 (24.6)	41 (26.7)	39.7 (27.2)	− 7.4 [− 23. 7,8.9]	0.3545	1	13.2 [− 3.1, 29.5]	0.1063	1	0.4455
F	Force	20	0.5 (0.3)	1 (0.9)	1.7 (0.9)	2.2 (1.9)	0.5 [− 0.3, 1.2]	0.1968	1	1.2 [0.5, 1.9]	0.0025	0.0733	0.9217
F	Force	50	1.5 (1.4)	1.5 (1.3)	4.4 (2.3)	3 (2)	− 0.7 [− 1.9, 0.5]	0.2383	1	2.2 [0.9, 3.4]	0.0013	0.046	0.2338
F	Force	80	5.3 (3.6)	2.5 (2.2)	8.1 (2.8)	4.4 (3.2)	− 3.3 [− 5.5, − 1.1]	0.005	0,1354	2.4 [0.2, 4.5]	0.0364	0.8744	0.6817
E	Torque	20	5.2 (3.6)	3.6 (2.4)	12.3 (7.2)	14.4 (12.2)	0.3 [− 4.4, 5]	0.8936	1	9 [4.3, 13.6]	0.0007	0.0249	0.424
E	Torque	50	6.1 (5.4)	10.5 (7.9)	22.1 (17.7)	35.7 (27.5)	9 [− 2.4, 20.4]	0.1152	1	20.6 [9.2, 32]	0.0012	0.0421	0.4081
E	Torque	80	22 (18.9)	15.8 (11)	49.6 (25.1)	40.4 (40.7)	− 7.7 [− 23.4, 8]	0.3187	1	26.1 [10.4, 41.8]	0.0024	0.0733	0.8482
E	Force	20	0.4 (0.2)	0.7 (0.5)	1.8 (1.4)	2.3 (1.4)	0.4 [− 0.3, 1.1]	0.2589	1	1.5 [0.8, 2.1]	0.0002	0.008	0.7418
E	Force	50	0.7 (0.4)	1.1 (0.8)	2.4 (1.5)	3.5 (2.3)	0.7 [− 0.2, 1.7]	0.1224	1	2.1 [1.1, 3]	0.0002	0.0075	0.4606
E	Force	80	1.9 (1)	1.9 (1)	3.5 (0.9)	5.1 (3.8)	0.8 [− 0.7, 2.2]	0.2846	1	2.4 [1, 3.8]	0.0024	0.0733	0.2684
R	Torque	20	2.2 (0.8)	5 (4.1)	12.8 (3.1)	9.4 (7.5)	− 0.3 [− 3.6, 2.9]	0.8268	1	7.5 [4.3, 10.7]	0.0001	0.0037	0.06
R	Torque	50	17.6 (16.8)	10.6 (7.4)	26.9 (22.1)	17.3 (9.2)	− 8.3 [− 16.7, 0.1]	0.0525	1	8 [− 0.4, 16.4]	0.0612	1	0.7464
R	Torque	80	52.2 (36.3)	22 (17.5)	52.4 (45.5)	26.1 (20.1)	− 28.2 [− 44.2, − 12.2]	0.0014	0,0485	2.2 [− 13.8, 18.1]	0.7813	1	0.8025
R	Force	20	0.3 (0.2)	0.8 (0.5)	1.2 (0.8)	1.8 (1)	0.6 [0.1, 1.1]	0.0287	0,7309	0.9 [0.4, 1.4]	0.0009	0.0328	0.8028
R	Force	50	1 (0.5)	1.8 (1.2)	3.3 (1.5)	4.8 (3)	1.2 [− 0.1, 2.4]	0.0605	1	2.7 [1.4, 3.9]	0.0002	0.0094	0.5397
R	Force	80	2.9 (1.7)	3 (1.8)	5.6 (2)	7.3 (4.1)	0.9 [− 0.8, 2.6]	0.2778	1	3.5 [1.8, 5.2]	0.0003	0.0115	0.3612
U	Torque	20	2.3 (1.3)	4.5 (3.4)	11.1 (6.9)	8.6 (7.3)	− 0.2 [− 4.1, 3.8]	0.9263	1	6.4 [2.5, 10.4]	0.0026	0.0738	0.2287
U	Torque	50	2.2 (0.6)	5.2 (2.8)	15.3 (11.5)	25.4 (14.1)	6.6 [0.8, 12.4]	0.0281	0,7309	16.6 [10.8, 22.4]	0	0.0003	0.2177
U	Torque	80	4.7 (6.1)	8.8 (3.1)	23.6 (16)	33.5 (21)	7.1 [− 2.1, 16.2]	0.1246	1	21.8 [12.6, 31]	0.0001	0.0031	0.5212
U	Force	20	0.3 (0.2)	0.7 (0.6)	1.5 (0.6)	2.5 (0.7)	0.7 [0.3, 1.1]	0.0018	0,0569	1.4 [1, 1.8]	0	0	0.139
U	Force	50	0.5 (0.3)	1.2 (1.2)	3 (1.9)	4.3 (1.8)	1 [− 0.1, 2]	0.0783	1	2.8 [1.7, 3.9]	0	0.001	0.5976
U	Force	80	0.6 (0.6)	2.9 (2.7)	4.7 (2.2)	4.9 (3)	1.2 [− 0.5, 2.9]	0.165	1	3 [1.3, 4.7]	0.0015	0.0495	0.2301

The column “Wire difference” shows the mean differences [95% confidence intervals] between measurements with and without K-wire, averaged over measurements with and without wrist loading. Similarly, the column “Loading difference” shows the mean differences [95% confidence intervals] between measurements with and without wrist loading, both averaged over measurements with and without K-wire. The two stages of one factor were averaged across both stages of the other factor. *P* values correspond to tests of the true mean differences being zero. Multiplicity-adjusted *P* values were calculated using the Bonferroni–Holm method across all tests for mean differences. The column “Interaction *P*” contains *P* values for the test of the null hypothesis, which was that the mean difference between the stages of one factor will be identical in both stages of the other factor

*Dir* direction, *F* flexion, *E* extension, *R* radialduction, *U* ulnarduction

### Flexion

Flexion of the wrist was guided to 54.5 ± 10.7° and to 54.9 ± 10.04° during loading. K-wire arthrodesis limited flexion to 42.5 ± 10.2° without loading and to 43.8 ± 12.1° with it. Wrist flexion from 20 to 80% ROM caused torque levels between the scaphoid fragments from 3.9 ± 3.4 to 33.9 ± 28.9 Nmm and force levels from 0.5 ± 0.3 to 5.3 ± 3.6 N. We observed a significant increase (adjusted *P* value, 0.0172) of 7.1 [95% confidence interval, 3.5, 10.6] Nmm in the mean torque at 20% ROM when the wrist was loaded. For all other ROM values, the corresponding increase in torque and force was not significant (adjusted *P* values: 1 for 50% and 1 for 80% wrist flexion in torque between the fragments; adjusted *P* values: 0.07 for 20%, 0.046 for 50%, and 0.87 for 80% ROM wrist flexion in force between the fragments). The highest average torque values within the scaphoid were measured at 80% of flexion in the unloaded (33.9 ± 28.9 Nmm) and loaded states (41.0 ± 26.7 Nmm). The highest average values of force within the scaphoid were measured at 80% of flexion in the unloaded (5.3 ± 3.6 N) and loaded states (8.1 ± 2.8 N).

### Extension

Wrist extension was guided to 77.6 ± 16.04° and to 71.46 ± 13.4° during loading. K-wire arthrodesis limited extension to 58.75 ± 18.8° without and to 52.4 ± 19° with wrist loading. Wrist extension without loading from 20 to 80% of ROM caused torque levels between the scaphoid fragments from 5.2 ± 3.6 to 22 ± 18.9 Nmm and force levels from 0.4 ± 0.2 to 1.9 ± 1 N. When the wrist was loaded, we observed a significant increase (adjusted *P* value, 0.025) of 9 Nmm [4.3, 13.6] in the mean torque at 20% ROM, a significant increase (adjusted *P* value, 0.042) of 20.6 [9.2, 32] Nmm in the mean torque at 50% ROM, a significant increase (adjusted *P* value, 0.008) of 1.5 [0.8, 2.1] N in the mean force at 20% ROM, and a significant increase (adjusted *P* value, 0.007) of 2.1 [1.1, 3] N in the mean force at 50% ROM. For all other values of ROM, the corresponding increase in torque and force by loading was not significant (adjusted *P* value, 0.07 for 80% wrist extension in torque between the fragments; adjusted *P* value, 0.07 for 80% ROM wrist extension in force between the fragments). Highest average values of torque within the scaphoid were measured at 80% of extension in the unloaded (22 ± 17.5 Nmm) and loaded states (49.6 ± 25.1 Nmm). Highest average values for force within the scaphoid were measured at 80% of extension in the unloaded (1.9 ± 1 N) and loaded states (3.5 ± 0.9 N).

### Radial abduction

Radial abduction of the wrist was guided to 23.3 ± 5.18° and to 24.9 ± 4.23° during loading. K-wire arthrodesis limited radial abduction to 17.52 ± 6.55° without and to 17.8 ± 6.52° with loading of the wrist. Radial abduction of the wrist without loading from 20 to 80% of ROM caused torque levels between the scaphoid fragments from 2.2 ± 0.8 to 52.2 ± 36.3 Nmm and force levels from 0.3 ± 0.2 to 2.9 ± 1.7 N. We observed a significant increase (adjusted *P* value, 0.0037) of 7.5 Nmm in the mean torque at 20% ROM by loading of the wrist. For all other values of ROM, the corresponding increase in torque was not significant.

When the wrist was loaded, we observed a significant increase (adjusted *P* value, 0.0328) of 0.9 [0.4, 1.4] N in the mean force at 20% ROM, a significant increase (adjusted *P* value, 0.0094) of 2.7 [1.4, 3.9] N in the mean force at 50% ROM, and a significant increase (adjusted *P* value, 0.0115) of 3.5 [1.8, 5.2] N in the mean force at 80% ROM. The highest average values of torque within the scaphoid were measured at 80% of radial abduction in the unloaded state (52.2 ± 36.3 Nmm) and in the loaded state (52.4 ± 45.5 Nmm). The highest average values of force within the scaphoid were measured at 80% of wrist radial abduction in the unloaded (2.9 ± 1.7 N) and loaded states (5.6 ± 2 N).

#### Ulnar abduction

Ulnar abduction of the wrist was guided to 31.37 ± 1.26° and to 31.5 ± 1.59° during loading. K-wire arthrodesis limited ulnar abduction to 27.77 ± 5.5° without and to 28.47 ± 7.9° with loading of the wrist. We observed a significant increase (adjusted *P* value, 0.0003) of 16.6 [10.8, 22.4] Nmm in the mean torque at 50% ROM and a significant increase (adjusted *P* value, 0.0031) of 21.8 [12.6, 31] Nmm in the mean torque at 80% ROM by loading of the wrist. We observed a significant increase (adjusted *P* value, 0) of 1.4 (1, 1.8) N in the mean force at 20% ROM, a significant increase (adjusted *P* value, 0.001) of 2.8 (1.7, 3.9) N in the mean force at 50% ROM, and a significant increase (adjusted *P* value, 0.05) of 3 [1.3, 4.7] N in the mean force at 80% ROM by loading of the wrist. For all other ROM values, the corresponding increase in torque was not significant. The highest average values of torque within the scaphoid were measured at 80% of ulnar abduction in the unloaded (4.7 ± 6.1 Nmm) and loaded states (23.6 ± 16 Nmm). Highest average values of force within the scaphoid were measured at 80% of wrist ulnar abduction in the unloaded (0.6 ± 0.6 N) and loaded states (4.7 ± 2.2 N). In summary, our results suggest that tendon loading significantly affects torque and force within the scaphoid during all moving directions, depending on ROM.

#### Direction of torque and lever force

Observation of the individual specimens demonstrated torque direction varying among the selected movement directions and within a given direction. This variation depended in part on the magnitudes of the deflection from 20 to 80% ROM, loading condition, and specimen. In extension with and without tendon loading, torque consistently supinated the proximal fragment (Table 3). With wrist flexion and radial duction, lever force acted in a radial direction. With wrist ulnar deviation, the force within the scaphoid was directed radially and plamarly; with wrist extension, we observed a tendency to a radiodorsal force direction (Supplementary Figure 2). Regardless of the direction of wrist movement, partial wrist arthrodesis caused a change in force direction, but without any detectable or predictable pattern (Supplementary Figure 3).

**Table 3:** Direction of torque due to different movements, loading conditions and k-wire blocking of the wrist

Specimen	***No k-wires***	***No k-wires***	***k-wires***	***k-wires***	***No k-wires***	***No k-wires***	***k-wires***	***k-wires***
	***No loading***	***Loading***	***No loading***	***Loading***	***No loading***	***Loading***	***No loading***	***Loading***
	**Flexion**	**Flexion**	**Flexion**	**Flexion**	**Extension**	**Extension**	**Extension**	**Extension**
**1**	pron	sup	sup	sup	pron	sup	pron	sup
**2**	sup	sup	sup	sup	sup	sup	pron	pron
**3**	sup	sup	sup	sup	sup	sup	sup	sup
**4**	pron	pro	pron	0	0	sup	sup	0
**5**	sup	sup	sup	sup	sup	sup	sup	sup
**6**	Pron	pron	sup	sup	sup	sup	sup	sup
**7**	pron	pron	0	pron	sup	sup	pron	0
**8**	sup	sup	sup	sup	sup	sup	sup	Sup
	**Radial**	**Radial**	**Radial**	**Radial**	**Ulnar**	**Ulnar**	**Ulnar**	**Ulnar**
**1**	pron	pron	pron	pron	0	0	sup	sup
**2**	sup	sup	sup	sup	sup	sup	sup	sup
**3**	sup	sup	sup	sup	sup	sup	sup	sup
**4**	pron	pron	pron	sup	0	sup	sup	sup
**5**	sup	sup	0	sup	sup	0	sup	sup
**6**	sup	sup	sup	sup	pron	pron	sup	sup
**7**	pron	0	pro	0	pron	pron	sup	0
**8**	sup	sup	sup	sup	sup	sup	pron	sup

## Discussion

The objective of this study was to determine wrist movement-generated torque and orthogonally acting lever force around the longitudinal scaphoid axis and the possible association of carpal bone size, wrist ROM, and surgical neutralisation of torque. Our hypothesis was that intercarpal K-wire fixation of the adjacent carpal bones would reduce torque and lever within the fractured scaphoid bone. We found that torque and lever force did not show a systematic dependence on scaphoid size, individual wrist ROM, or deviations in the sensor versus the anatomic axis. In addition, K-wire fixation yielded no significant changes in average torque and lever force (except radial abduction) values. Other than wrist extension for torque and wrist flexion for lever force, the directions of the developed torque and lever force, respectively, were not predictable.

Here, we used an intraosseous sensor to examine isometric torque through the longitudinal axis of the scaphoid. Slade et al. suspected that the scaphoid is subjected to rotatory forces throughout the wrist ROM and confirmed their hypothesis that torque is generated within the scaphoid during wrist motion [4]. End-range wrist motion also exacerbates torque. We identified that the level of torque and lever force depends on the direction and extent of wrist motion and on whether the wrist is loaded. Our findings regarding torque along the anatomical scaphoid axis explain earlier reported interfragmentary rotation within the scaphoid bone [8–10]. Using three-dimensional image-matching technology, Oka et al. showed rotation of the proximal pole in the supination direction in unstable fractures that were localised distally to the scaphoid apex [11]. Although Smith et al. identified rotation, compression, or distraction, the primary forces acting on the distal scaphoid were those that displaced the scaphoid fracture in a flexed and pronated position [10]. Forces and torque measured in our study by wrist motion would conceivably result in a humpback deformity of the scaphoid body.

Our results cannot be directly compared to those of Fortis et al., who used rosette strain gauges to investigate how wrist motion influences the magnitude of the specific strain and compression on the intact scaphoid [12]. Other authors assumed a three-dimensional alignment of forces based on fundamental movement analyses of the wrist [8]. These analyses examine force distribution across individual compartments of the wrist with pressure-sensitive conductive rubber, a rigid body spring modelling technique, or finite element analysis [13–16].

Centrally placed scaphoid screws can improve construct stiffness compared with eccentrically placed scaphoid screws [5]. Indeed, several studies have examined stabilisation of fractures with a single traction screw, including interfragmentary compression with screws across a simulated scaphoid fracture [17, 18]. In addition, various techniques to assess motion of scaphoid fragments have been applied in cadaver studies addressing the stability of osteosyntheses arising from cyclic loading [5, 19, 20]. We also assumed for our sensor that a centrally placed screw would enhance construct stiffness. After mathematical angle alignment of the sensor axis onto the central axis of the scaphoid, however, we could not explain the measurement fluctuations caused by the diverse positions of the sensor in the scaphoid between each specimen. Presumably these fluctuations in direction and dimension of torque and force depend on the alignment of the sensor to the main pulling direction of the wrist crossing tendons, which again depend on the individual soft tissue and bony wrist structures and positional differences.

A uniform direction of force within a sector of 90° radiopalmar direction of coronal scaphoid plane could be determined only in wrist flexion. Conversely, a uniform direction of torque could be determined only in extension of the wrist; thus, we can draw no conclusions regarding the rotational direction in which a scaphoid osteosynthesis screw provides more stability. In our opinion, the rigidity of a scaphoid osteosynthesis using a single bone screw will prevent more lever forces along the longitudinal axis than rotational torques generated from bypassing the lever forces.

To minimise the force of the investigator, torque was examined only at 20%, 50%, and 80% of the respective ROM. Because high torque values > 0.1 Nm were measured in 80% of full ROM in flexion, radial duction, and extension in some of the individuals, even higher torques can be expected and must be withstood by single-screw osteosyntheses in vivo because patients will perform movements to the full end-ROM. In our investigation, from 20% extension to 50% flexion, we measured low torque values below 25% of average torque at 80% of ROM. Based on these results, we cannot recommend a preferred movement direction. Rather, as clinical postoperative guidelines after screw osteosyntheses, we suggest a defined limited ROM from 20% extension and radial abduction to 50% flexion. Important parameters affecting torque in the scaphoid involve individual wrist factors. However, we could not determine that the varying sizes of the scaphoid and the longitudinal axis affected the fluctuations in torque and lever force between the specimens during wrist motion. In addition, K-wire intercarpal stabilisation did not reduce torque magnitude between the two fragments. Our current data do not allow us to provide further details to clarify the cause of the varying torque and force magnitudes between the specimens.

## Limitations of the study

As studied on long bones, evidence is limited on the biomechanics involved in fracture healing of the scaphoid bone [21, 22]. During pilot testing, we found that wrist motion could not be controlled and was not sufficiently reproducible with the initial tension of the tendons specified in the experiments. To simulate in vivo movement, we used a device that passively drew the wrist into all four motions without pulling on the tendons. We are aware that pure passive motion without wrist loading in cadavers does not correspond to in vivo loading. Grasping movement better approximates the everyday loading at the wrist by generating co-contraction of the flexor and extensor tendons and stabilisation of the tare weight under wrist movement. In previous reports, tendon loading was performed at 98 N [23, 24]. However, in our pilot study, we determined that wrist loading of 70 N was appropriate to simulate an in vivo condition of holding a 0.5-kg bottle in supination. Biomechanical changes caused by reduced viscosity from specimen drying were assumed. Therefore, the specimens were constantly moistened with isotonic saline. Resulting changes in the dissected and sutured capsule and ligaments cannot be ruled out.

In addition, with our experimental setup, we could not identify scaphoid size as an influencing factor. Other anatomical variations such as ligamentous laxity, scaphoid shape, and intercarpal arrangement cannot be investigated with our experimental approach but are likely to cause variations in measured forces.

The design and usage of the sensor also involved several limitations. The in vitro application differed from the calibrating procedure because only one end was fixed in a chuck for lever force measurement. For torque calibration, one end was fixed in every direction, and the other end was stabilised against the lever force but kept free in rotation to avoid combined torque and lever force. In vitro, both ends were fixed to ligament-stabilised but mobile bone fragments. The result was a combination of flexibility in rotation and bending along the sensor axis. Discrepancy in necessary tendon-pulling forces to achieve comparable wrist motion did not allow for force-controlled wrist motion in our experiments.

Because of a lack of sensor surface area, we used half-bridges for force measurement. For the sensor construction, Wheatstone full-bridges would have been best not only for torque but also for lever force measurement to eliminate simultaneous upsetting force and thermal loading orthogonal to the sensor axis.

In addition to interrupted circulation, prolonged healing after single-screw osteosynthesis might arise because of an existing instability occurring during wrist loading and motion [11, 25]. Wrist motion leads to torque and lever force along the longitudinal axis of the scaphoid. Forces produced by ligament and tendon traction and pressure must be neutralised in the treatment of unstable scaphoid fractures.

## Conclusion

Our results add to understanding of the biomechanics of the scaphoid and indicate the need for greater fixation rigidity in fracture cases and non-unions. These findings serve as a basis for additional experiments to examine the level of torque and lever force that a screw-fragment construct should be able to withstand.

## Supplementary information

**Additional file 1: Supplementary Figure 1**: Force within the scaphoid at 20%, 50%, and 80% of wrist ROM (flexion, extension, radial abduction, ulnar abduction) in the unloaded and loaded states with and without K-wire stabilisation.

**Additional file 2: Supplementary Figure 2**: Lever force direction between the scaphoid fragments during wrist motion at 80% of ROM of any wrist movement direction.

**Additional file 3: Supplementary Figure 3**: Lever force direction between the scaphoid fragments during wrist motion at 80% of ROM of any wrist movement. In this condition, the wrist was partially blocked by K-wires.

## Data Availability

The datasets supporting the conclusions of this article are provided within the article.

## Abbreviations

ROM: Range of motion
