# Supplementary figures and images for: Wrist movements induce torque and lever force in the scaphoid: an ex vivo study
Source: J Orthop Surg Res. 2020 Aug 31;15:368. doi: 10.1186/s13018-020-01897-y (PMC7457810; doi:10.1186/s13018-020-01897-y)

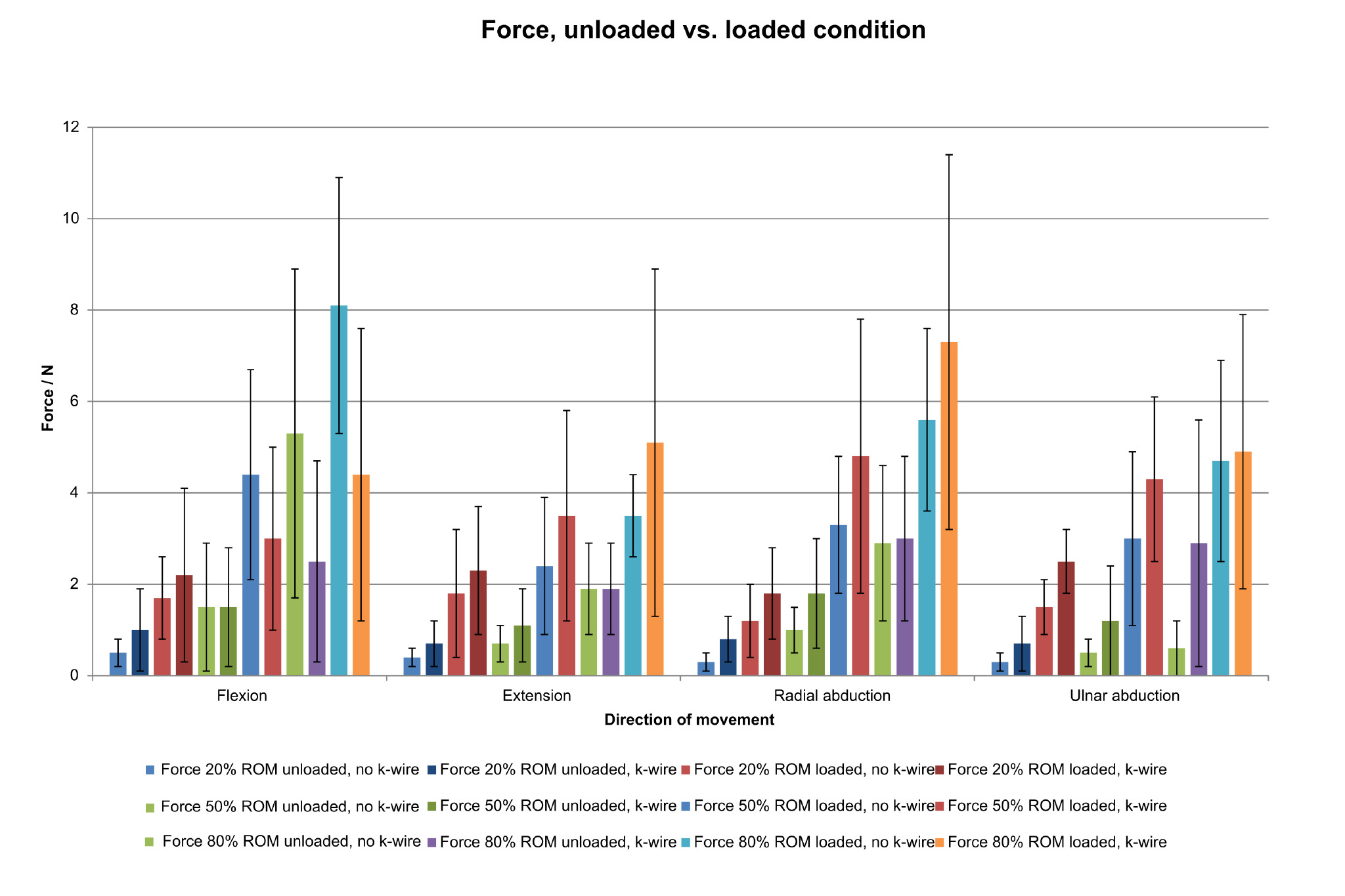

Supplement: Supplementary file 1 — Additional file 1: Supplementary Figure 1: Force within the scaphoid at 20%, 50%, and 80% of wrist ROM (flexion, extension, radial abduction, ulnar abduction) in the unloaded and loaded states with and without K-wire stabilisation. [file 13018_2020_1897_MOESM1_ESM.jpg]

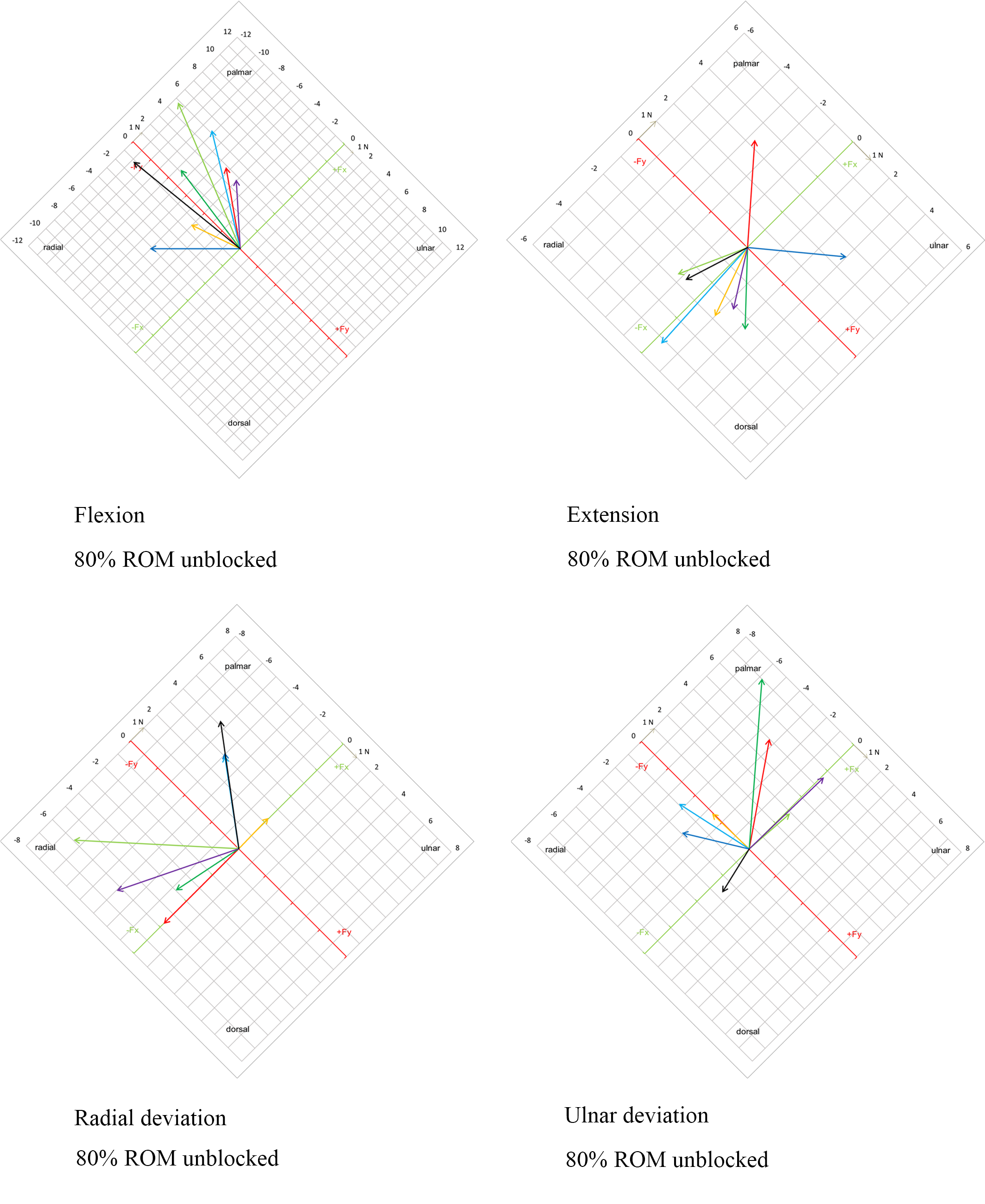

Supplement: Supplementary file 2 — Additional file 2: Supplementary Figure 2: Lever force direction between the scaphoid fragments during wrist motion at 80% of ROM of any wrist movement direction. [file 13018_2020_1897_MOESM2_ESM.tiff]

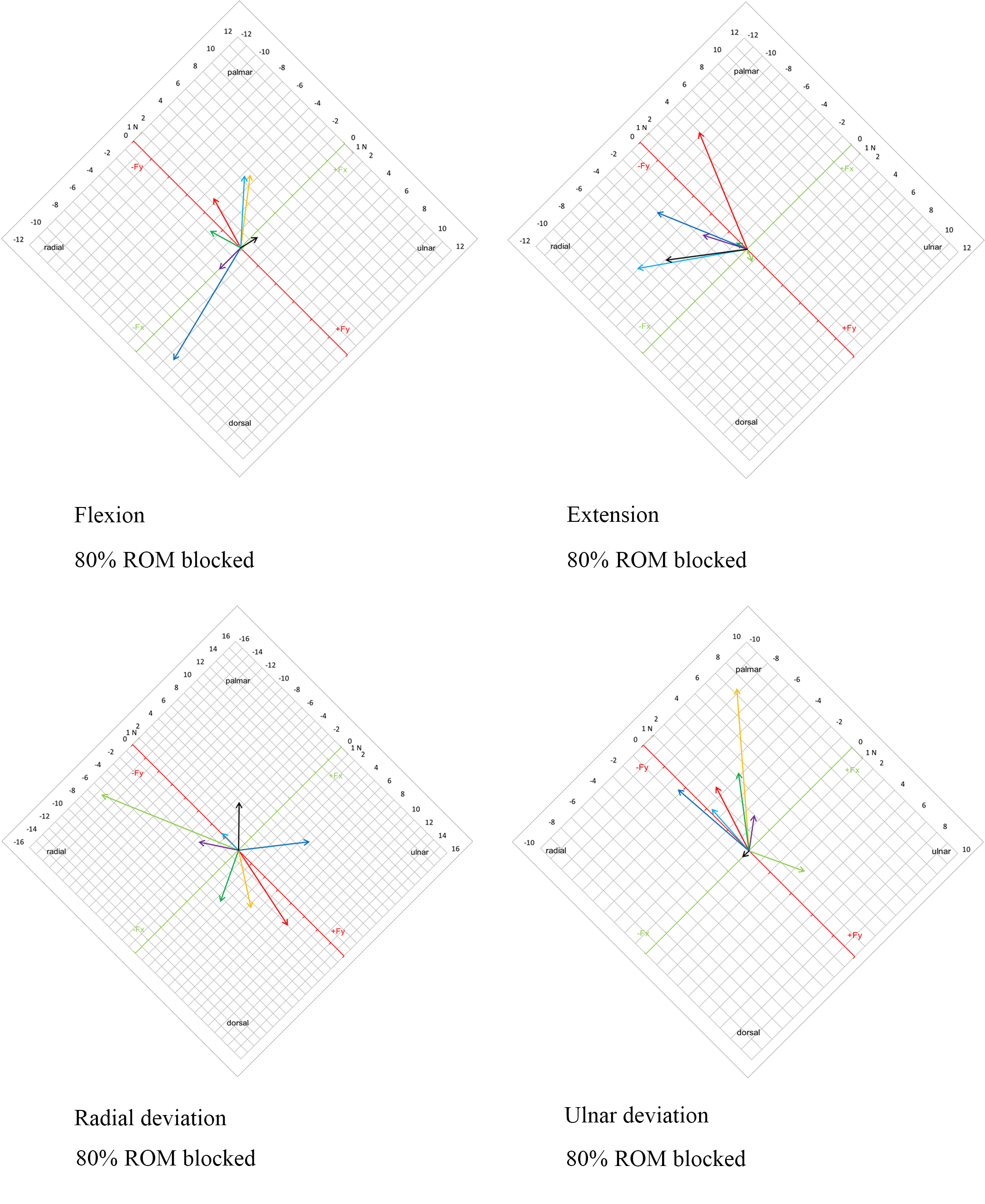

Supplement: Supplementary file 3 — Additional file 3: Supplementary Figure 3: Lever force direction between the scaphoid fragments during wrist motion at 80% of ROM of any wrist movement. In this condition, the wrist was partially blocked by K-wires. [file 13018_2020_1897_MOESM3_ESM.tiff]
